# Frailty and treatment outcome in advanced gastro-oesophageal cancer: An exploratory analysis of the GO2 trial

**DOI:** 10.1016/j.jgo.2021.12.009

**Published:** 2022-04

**Authors:** Jessica Pearce, Daniel Swinson, David Cairns, Sherena Nair, Mark Baxter, Russell Petty, Matt Seymour, Peter Hall, Galina Velikova, GO2 trial investigators

**Affiliations:** a Leeds Institute of Medical Research at St James', University of Leeds, Woodhouse, Leeds, LS2 9JT, UK; b Leeds Teaching Hospitals NHS Trust, Beckett Street, Leeds LS9 7TF, UK; c Leeds Cancer Research UK Clinical Trials Unit, Leeds Institute of Clinical Trials Research, University of Leeds, Leeds LS2 9JT, UK; d Division of Molecular and Clinical Medicine, Ninewells Hospital and Medical School, University of Dundee, Nethergate, Dundee DD1 4HN, UK; e Tayside Cancer Centre, Ninewells Hospital and Medical School, NHS Tayside, James Arrott Dr, Dundee DD2 1SG, UK; f Cancer Research UK Edinburgh Centre, MRC Institute of Genetics & Molecular Medicine, The University of Edinburgh, Western General Hospital, Crewe Road South, Edinburgh EH4 2XR, UK

**Keywords:** Frailty, Geriatric oncology, Oncogeriatrics, Gastro-oesophageal cancer, GCA, Geriatric assessment

## Abstract

**Introduction:**

Research into the optimal management of frail patients with cancer is limited and treatment decision-making in this cohort can be difficult. A number of measures have been developed to assess frailty, but few studies explore the correlation between frailty measures and cancer treatment outcomes.

**Methods:**

This retrospective cohort study is an exploratory analysis of the GO2 randomised controlled trial. GO2 recruited both older and frail younger patients commencing first-line palliative chemotherapy for advanced gastro-oesophageal (aGO) cancer. This analysis aims to explore the correlation between baseline frailty and treatment outcome. Baseline frailty measures were derived from clinical data and included ECOG Performance Status (PS), the GO2 Frailty Score (GO2FS), Geriatric-8 (G8), Cancer and Aging Research Group (CARG) toxicity score and a ‘modified’ Rockwood Clinical Frailty Scale (mCFS). Novel patient-centred composite measure Overall Treatment Utility (OTU) was the primary endpoint. Ordinal logistic regression was undertaken to give odds ratios for poor vs good/intermediate OTU. Secondary endpoints were progression-free and overall survival. Models were adjusted for age, sex, histology, metastases, Trastuzumab and renal/hepatic function.

**Results:**

In GO2, 514 patients were randomised between three chemotherapy dose-levels; all of these patients were assessed for OTU and are included in this analysis. Worse GO2FS, mCFS and G8 scores all had a statistically significant association with poor (vs good/intermediate) OTU, progression and death, which persisted after adjustment. Adjusted odds ratios for poor OTU amongst those with the worst GO2FS and mCFS and best G8 scores were as follows: 1.85 (95% confidence interval [CI] 1.20–2.88) for GO2FS ≥3 (‘severely frail’), 1.72 (1.19–2.50) for mCFS 5+ (‘frail’) and 0.57 (0.32–1.00) for G8 > 14 (‘normal’). Worse ECOG PS and CARG scores did not have a statistically significant association with poor OTU/progression/death.

**Conclusion:**

In this study, frailty identified via GO2FS, mCFS and G8 conveyed a statistically significant increased risk of worse treatment outcome in older and frail younger patients with aGO cancer. Frailty assessment provides information over and above PS and should be integrated alongside routine assessments in research and clinical practice. In the absence of prospective data, frailty measures can be derived retrospectively to build the evidence base around optimal care of frailer patients.

## 1 Introduction

Frailty due to older age or co-morbidities is common in patients with cancer and is associated with an increased risk of treatment complications [1]. Despite this, many clinical trials exclude older and frail younger patients, so evidence to guide optimal management is lacking. Eastern Cooperative Oncology Group (ECOG) Performance Status (PS) is the most widely used tool for assessing fitness for systemic anticancer therapy (SACT), but lacks granularity [2] and a number of criticisms have been highlighted [3]. The Rockwood Clinical Frailty Scale [4] (CFS) is a global assessment of fitness which has extensive evidence of correlation with outcome in a range of acute and outpatient settings, and has been adopted for use by the NHS Specialised Clinical Frailty Network (SCFN) in the United Kingdom (UK) [5]. It has also been proposed as an alternative to ECOG PS [6,7] but remains largely untested in the oncology setting.

Frailty screening tools to guide intervention and management of older cancer patients recommended by ASCO guidelines [8] include the Geriatric-8 (G8) [9] and Vulnerable Elders Survey (VES)-13 [10], which have both been independently associated with adverse outcomes in older patients with cancer receiving chemotherapy. Additionally, two toxicity prediction tools are recommended which have been demonstrated to provide estimates of risk of significant (grade ≥ 3) chemotherapy toxicity in older patients with cancer; Cancer and Aging Research Group (CARG) [11] and Chemotherapy Risk Assessment Scale for High-Age Patients (CRASH) [12].

**Fig. 1:**
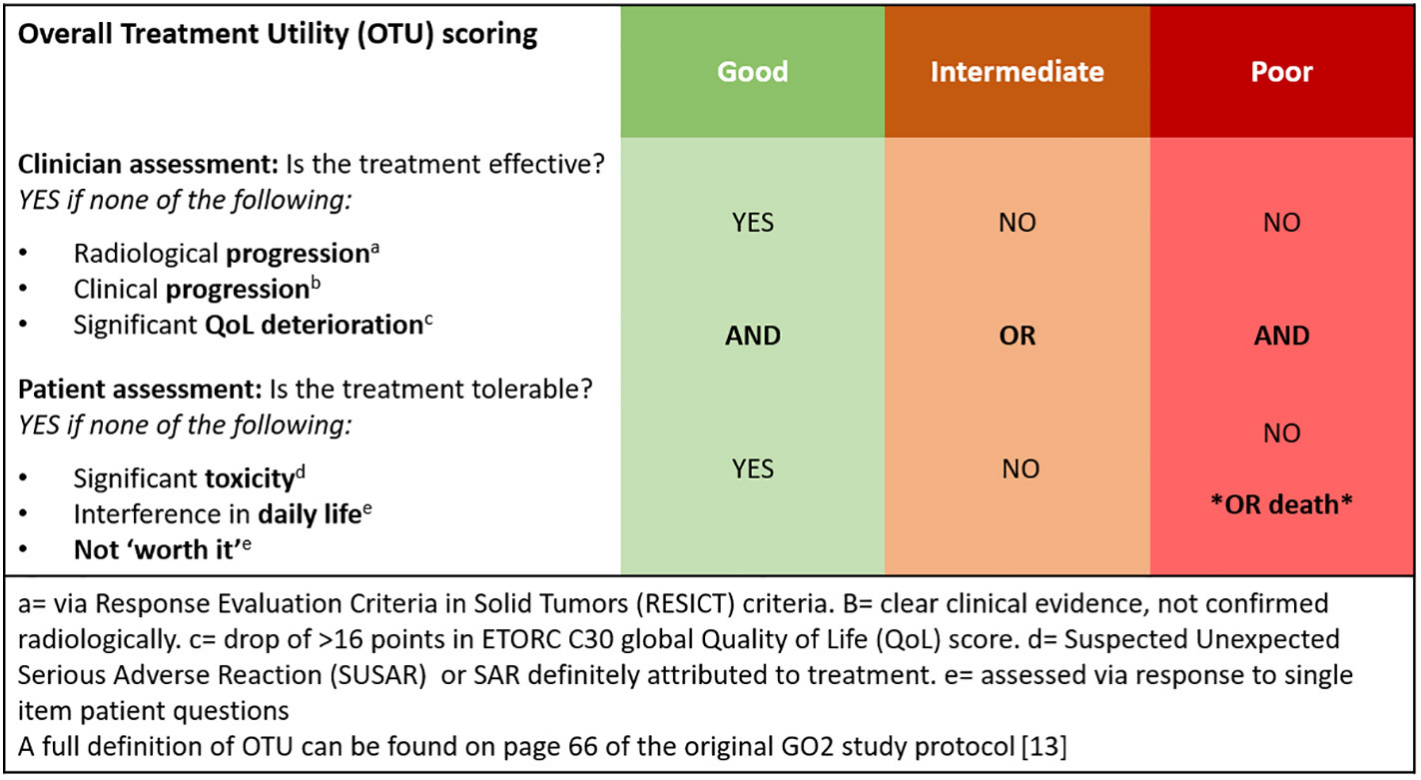
Overall treatment utility (OTU) scoring in the GO2 trial.

The aim of this study is to explore the correlation between baseline frailty measures and global treatment outcomes in older and frail younger patients with advanced gastro-oesophageal (aGO) cancer in the GO2 trial. The GO2 trial is a phase III randomised-controlled trial which sought to optimise chemotherapy dosing for older and frail younger patients with aGO cancer, and established 60% duplet chemotherapy as a new standard of care for these patients [13]. It also incorporated the novel patient-centred composite end-point Overall Treatment Utility (OTU) as a global measure of treatment outcome. OTU is categorised as Good, Intermediate or Poor by combining a range of factors particularly relevant to frail and older patients with advanced cancer, including clinical efficacy, toxicity, tolerability, and the patient's own assessment of treatment value (see Fig. 1). GO2 is one of the largest studies prospectively exploring the treatment of older and frail younger patients, and extensive baseline data collected through geriatric assessment (GA) provides a unique opportunity to explore the correlation between frailty and treatment outcomes.

## 2 Methods

A retrospective cohort study was undertaken as an exploratory analysis of the GO2 randomised controlled trial database.

### 2.1 Participants

The GO2 study recruited patients from 52 sites across the UK with aGO cancer (carcinoma of the oesophagus, gastroesophageal junction or stomach) who were deemed unsuitable for full-dose combination chemotherapy due to advanced age and/or frailty. Further details about eligibility and recruitment can be found in the publication of the primary analysis and trial protocol [13]. All patients in the CHEMO-INTENSITY pathway (i.e. those being randomised between different dose levels of chemotherapy, rather than 60% chemotherapy vs best supportive care) were included in this analysis (514 patients).

### 2.2 Frailty measures

A list of potential frailty measures was obtained through literature review and expert opinion of elderly medicine colleagues. The GO2 database was then interrogated to ascertain which measures were derivable from the baseline data. Baseline data included routine demographic data and GA, including the following questionnaires: G8 and Instrumental Activities of Daily Living (IADL) (both completed by nurse alongside patient) and two self-completed quality of life questionnaires (European Organisation for Research and Treatment of Cancer (EORTC) QLQ-C30 and EQ-5D-3L) as well as assessment of impairments in 9 frailty domains which informed the pre-determined GO2 frailty score (GO2FS, further described in Table 1).

**Table 1:** Derivable frailty measures selected for evaluation: rationale, source and categorisation.

Frailty measure	Rationale for selection	Source of data	Type of data and categorisation
ECOG Performance Status	Used routinely in the assessment of fitness for SACT	Clinician-assessed prospectively	Categorical: scores 0, 1, 2 or > 2 – as collected in GO2 study [13]
GO2 Frailty Score [13]	Pre-determined geriatric assessment-based frailty measure for the GO2 study based on published literature [1]	Measured prospectively by assessing impairment (yes/no) in 9 frailty domains: weight loss, mobility, falls, neuropsychiatric, physical functioning, social functioning, mood, fatigue, and polypharmacy	Numeric: Number of domains with impairments, out of 9 Categorical: Not frail (0–1/9 domain impaired), Mildly frail (2/9 domains impaired), Severely frail (≥3/9 domains impaired) - as categorised in original publication [13]
‘Modified’ Clinical Frailty Scale	Global assessment of frailty, potentially easy to undertake but not yet tested in this setting	7-point scale based on the Rockwood Clinical Frailty Scale [4]. Derived retrospectively via an algorithm from questionnaire data regarding patients' function, fatigue and impact of symptoms/disease (supplement 1).	Numeric: Score 1–7 Categorical: Fit (1–2), Pre-frail (3–4), Frail (5+) – guided by feedback expert review and findings of statistical analysis
Geriatric-8[9]	Frailty screening tool recommended by ASCO consensus guidelines [8]	8-item questionnaire collected prospectively encompassing food intake, weight loss, mobility, neuropsychological problems, BMI, polypharmacy, self-rated health and age.	Numeric: Total score of 0–17 (lower scores align with adverse features) Categorical: >14 (‘normal’), ≤14 (‘abnormal’) – based on threshold to refer for GCA [9]
Cancer and Aging Research Group toxicity score [11]	Toxicity prediction tool recommended by ASCO consensus guidelines [8]	11 items encompassing age, cancer type, chemotherapy schedule, functioning, anaemia and kidney function. Derived retrospectively.	Numeric: Total score of 0–19 (higher scores reflecting adverse features). Categorical: Low risk (0–5), Medium risk (6–9), High risk (10–19) - based on published literaure [11]

The derivable frailty measures selected for evaluation in this analysis are shown in Table 1.

### 2.3 ‘Modified’ clinical frailty scale (mCFS)

Rockwood CFS is a simple global assessment of fitness designed to be undertaken by a clinician. Patients are scored 1–9 based on descriptors outlining functional capabilities, fatigue and impact of symptoms [4]. Rockwood CFS was not collected prospectively in GO2, but given its exceptional simplicity and growing use in the UK [5], we were keen to derive a proxy measure for this study from the baseline questionnaire data. As a piece of exploratory work, authors JP (oncology trainee), DS (oncology consultant) and SN (elderly medicine consultant) drafted an algorithm to derive a ‘modified’ CFS based on selected questions and responses in the baseline assessments/questionnaires which aligned closest with the Rockwood CFS scores and descriptors. The algorithm was independently reviewed by two elderly medicine consultants for face validity and amended in line with feedback until a ‘best fit’ for deriving CFS from the available data was agreed (supplementary appendix 1). Rockwood CFS is a 9-point scale (1 ‘Very Fit’ – 9 ‘Terminally Ill’); the modified CFS (mCFS) was abbreviated to a 7-point scale (1 ‘Very Fit’ - 7 ‘Living with Severe Frailty’), as patients with the extreme degree of frailty reflected by a Rockwood CFS >7 would not have been considered eligible for GO2 on fitness grounds, and additionally because the baseline questionnaire data was unable to reliably identify patients whom fulfilled the descriptors for Rockwood CFS scores of 8 (‘Living with very severe frailty’) or 9 (‘Terminally ill’).

### 2.4 Outcomes

Overall Treatment Utility (OTU), measured at 9 weeks, was selected as the primary outcome for this analysis due to its ability to provide a global, holistic measure of treatment outcome [14] (see Fig. 1). Secondary outcomes are progression-free and overall survival. Study endpoints are fully defined in the trial protocol and statistical analysis plan [13] and conform to the joint EORTC/International Society of Geriatric Oncology (SIOG) Statement [15].

### 2.5 Statistical analyses

The coding platform Rstudio was used to calculate frailty scores and undertake statistical analyses. OTU comparisons used ordinal logistic regression to estimate odds ratios (ORs) for a poor vs good or intermediate OTU with 95% confidence intervals (CIs). Cox proportional hazards (CPH) regression [16] were used to estimate hazards ratios (HRs) and 95% CIs and Kaplan-Meier methods [17] were used to estimate survivor functions for time-to-event endpoints. Numeric and grouped data was used for the ordinal logistic regression, and grouped data for CPH regression. Analyses for all frailty measures were treated and reported in the same way, for consistency and to allow comparison between ORs and HRs for each frailty measure. Groupings and rationale for GO2FS, CARG and G8 are outlined in Table 1. Models were adjusted for potential confounders: age group, sex, histology, distant metastases, planned use of Trastuzumab and dose reduction due to renal or hepatic function. Performance status was also adjusted for in additional analyses of GO2FS, mCFS, G8 and CARG, to provide an indication as to whether these scores add information on top of the current standard of care in fitness assessment (PS), but was not included in the final adjustment for consistency and due to concerns regarding co-linearity between frailty measures and PS. Variable Inflation Factors (VIF) testing was undertaken on each model to look for multi-collinearity.

### 2.6 Ethics

Ethical approval for the GO2 trial was provided by the UK National Research Ethics Service and written informed consent was obtained from all patients.

## 3 Results

### 3.1 Participants

In GO2, 514 patients were randomised between three chemotherapy dose-levels; all of them underwent baseline GA, were assessed for OTU, and are included in this analysis. No patients were lost to follow-up and the median follow-up time was 12.6 months [IQR 11.9, 12.9]. Baseline patient characteristics are shown in Table 2. Frailty measures, stratified by treatment outcomes across all treatment allocations are summarised in Table 3.

**Table 2:** Baseline patient characteristics.

X.	Total
	(*N* = 514)
Age (years)	
Median [Min, Max]	76.0 [51.0, 96.0]

Sex	
Male	385 (74.9%)
Female	129 (25.1%)

Histology	
Squamous	57 (11.1%)
Non-squamous	457 (88.9%)

Metastases	
Yes	347 (67.5%)
No	167 (32.5%)

Planned use of Trastuzumab	
Yes	28 (5.4%)
No	486 (94.6%)

Dose reduction due to renal/hepatic function	
Yes	45 (8.8%)
No	469 (91.2%)

**Table 3:** Percentage of all patients within each frailty score category, whole cohort and stratified by treatment outcome.

		OTU @ 9 weeks			Progressed		Died	
X.	Total	Good	Int.	Poor	No	Yes	No	Yes
	(N = 514)	(*N* = 196)	(*N* = 149)	(*N* = 169)	(*N* = 76)	(*N* = 438)	(*N* = 141)	(*N* = 373)
ECOG PS								
0	72 (14.0%)	33 (16.8%)	21 (14.1%)	18 (10.7%)	9 (11.8%)	63 (14.4%)	17 (12.1%)	55 (14.7%)
1	279 (54.3%)	106 (54.1%)	84 (56.4%)	89 (52.7%)	47 (61.8%)	232 (53.0%)	88 (62.4%)	191 (51.2%)
2	148 (28.8%)	54 (27.6%)	37 (24.8%)	57 (33.7%)	19 (25.0%)	129 (29.5%)	32 (22.7%)	116 (31.1%)
3+	13 (2.5%)	3 (1.5%)	6 (4.0%)	4 (2.4%)	1 (1.3%)	12 (2.7%)	4 (2.8%)	9 (2.4%)
Missing	2 (0.4%)	0 (0%)	1 (0.7%)	1 (0.6%)	0 (0%)	2 (0.5%)	0 (0%)	2 (0.5%)

GO2FS								
Not frail (0–1)	93 (18.1%)	45 (23.0%)	25 (16.8%)	23 (13.6%)	16 (21.1%)	77 (17.6%)	31 (22.0%)	62 (16.6%)
Slightly frail (2)	121 (23.5%)	53 (27.0%)	32 (21.5%)	36 (21.3%)	22 (28.9%)	99 (22.6%)	41 (29.1%)	80 (21.4%)
Severely frail (3+)	298 (58.0%)	98 (50.0%)	91 (61.1%)	109 (64.5%)	38 (50.0%)	260 (59.4%)	69 (48.9%)	229 (61.4%)
Missing	2 (0.4%)	0 (0%)	1 (0.7%)	1 (0.6%)	0 (0%)	2 (0.5%)	0 (0%)	2 (0.5%)

mCFS								
Fit (1–2)	174 (33.9%)	80 (40.8%)	48 (32.2%)	46 (27.2%)	35 (46.1%)	139 (31.7%)	63 (44.7%)	111 (29.8%)
Pre-Frail (3–4)	110 (21.4%)	38 (19.4%)	38 (25.5%)	34 (20.1%)	15 (19.7%)	95 (21.7%)	25 (17.7%)	85 (22.8%)
Frail (5+)	230 (44.7%)	78 (39.8%)	63 (42.3%)	89 (52.7%)	26 (34.2%)	204 (46.6%)	53 (37.6%)	177 (47.5%)

G8								
>14 (‘normal’)	42 (8.2%)	18 (9.2%)	19 (12.8%)	5 (3.0%)	9 (11.8%)	33 (7.5%)	18 (12.8%)	24 (6.4%)
≤14 (‘abnormal’)	458 (89.1%)	171 (87.2%)	127 (85.2%)	160 (94.7%)	63 (82.9%)	395 (90.2%)	119 84.4%)	339 (90.9%)
Missing	14 (2.7%)	7 (3.6%)	3 (2.0%)	4 (2.4%)	4 (5.3%)	10 (2.3%)	4 (2.8%)	10 (2.7%)

CARG								
Low risk (0–5)	41 (8.0%)	19 (9.7%)	13 (8.7%)	9 (5.3%)	5 (6.6%)	36 (8.2%)	9 (6.4%)	32 (8.6%)
Medium risk (6–9)	222 (43.2%)	89 (45.4%)	61 (40.9%)	72 (42.6%)	28 (36.8%)	194 (44.3%)	57 (40.4%)	165 (44.2%)
High risk (10–19)	215 (41.8%)	73 (37.2%)	66 (44.3%)	76 (45.0%)	35 (46.1%)	180 (41.1%)	63 (44.7%)	152 (40.8%)
Missing	36 (7.0%)	15 (7.7%)	9 (6.0%)	12 (7.1%)	8 (10.5%)	28 (6.4%)	12 (8.5%)	24 (6.4%)

Abbreviations: OTU = overall treatment utility, ECOG = Eastern Cooperative Oncology Group, PS Performance Status, GO2FS = GO2 Frailty Score, mCFS = ‘modified’ Clinical Frailty Scale, G8 = Geriatric-8, CARG = Cancer and Aging Research Group.

### 3.2 Odds ratios for poor OTU

Odds ratios for numeric and categorical frailty measure scores, before and after adjustment, are displayed in Table 4.

**Table 4:** Odds ratios poor vs good/intermediate Overall Treatment Utility (measured at 9 weeks) by frailty score (numeric) and category (for each frailty measure).

		Overall treatment utility Odds ratio (OR) for poor vs good/intermediate OTU, calculated via ordinal logistic regression	
Score		Unadjusted OR (CI)	Adjusted ^a^ OR (CI)
PS score	numeric^b^	1.25 (0.99, 1.56)	1.25 (0.99, 1.58)
PS category	0	1	1
	1	1.38 (0.86, 2.25)	1.40 (0.86, 2.28)
	2	1.65 (0.98, 2.81)	1.65 (0.97, 2.84)
	>2	1.83 (0.65, 5.25)	1.88 (0.64, 5.52)
GO2FS score	numeric	**1.18 (1.06, 1.33)**	**1.19 (1.06, 1.33)**^**c**^
GO2FS category	Not frail (0–1)	1	1
	Frail (2)	1.23 (0.74, 2.04)	1.23 (0.74, 2.06)
	Sev frail (≥3)	**1.84 (1.19, 2.85)**	**1.85 (1.20, 2.88)**^**c**^
mCFS score	numeric	**1.14 (1.02, 1.26)**	**1.14 (1.02, 1.27)**
mCFS category	Fit (1–2)	1	1
	Pre-frail (3–4)	1.43 (0.92, 2.22)	1.45 (0.94, 2.26)
	Frail (5+)	**1.72 (1.19, 2.49)**	**1.72 (1.19,2.50)**^**c**^
G8 score	numeric	**0.91 (0.85, 0.97)**	**0.91 (0.85–0.97)**^**c**^
G8 category	≤14 (‘abnormal’)	1	1
	>14 (‘normal’)	0.58 (0.32, 1.00)	0.57 (0.32, 1.00)
CARG score	numeric	1.05 (0.99, 1.11)	**1.07 (1.00, 1.13)**
CARG toxicity risk category	Low (0–5)	1	1
	Med (6–9)	1.43 (0.78, 2.67)	1.63 (0.84, 3.20)
	High (10–19)	1.75 (0.95, 3.27)	**2.10 (1.07, 4.17)**^**b**^

Statistically significant results in **bold**. a = adjusted for: age group, sex, histology, presence of metastases, planned use of trastuzumab, dose reduction due to renal or hepatic dysfunction. b = PS >2 was treated as 3 for the purpose of numeric analysis. c = significant after also adjusting for PS.

Abbreviations: OTU = overall treatment utility, PS = Performance Status, GO2FS = GO2 Frailty Score, mCFS = ‘modified’ Clinical Frailty Scale, G8 = Geriatric-8, CARG = Cancer and Aging Research Group.

Performance status was not associated with a statistically significant increased risk of poor OTU, with an adjusted odds ratio of 1.25 (0.99–1.58) in the numeric analyses.

Worse GO2FS, mCFS and G8 scores on the other hand were associated with poor OTU, with adjusted odds ratios of 1.19 (1.06–1.33), 1.14 (1.02–1.27) and 0.91 (0.85–0.97) respectively in the numeric analyses. This suggests that with each point increase in GO2FS and mCFS, the risk of a poor OTU increases by 18% and 14% respectively on the log odds scale. In categorical analyses, the highest GO2FS and mCFS scores are associated with a risk of poor OTU which increased by 85% (for GO2FS ≥3) and 72% (for mCFS 5+), respectively, though intermediate scores did not demonstrate a significant association. Conversely, with each point increase in G8, the risk of a poor OTU falls by 9%. However, the highest G8 scores were not significantly associated with a risk of a poor OTU in this analysis, with an adjusted odds ratio of 0.57 (0.32–1.00 [rounded down]) for G8 > 14.

CARG score was not significantly associated with risk of poor OTU pre-adjustment, but was after adjustment with an odds ratio of 1.07 (1.00 [rounded down] -1.13) in the numeric analyses and 2.10 (1.07–4.17) for the high-risk category (CARG score 10–19).

### 3.3 Hazard ratios for progression and death

Cox proportional hazards regression estimated HRs for progression and death respectively, adjusted and unadjusted HRs are displayed in Table 5. Overall survival curves stratified by each frailty measure are available in Supplementary Appendix 2.

**Table 5:** Hazard ratios for progression and death (respectively) within the trial period, by frailty category (for each frailty measure).

		Survival outcomes Hazard ratios (HR) for progression and death respectively, calculated via cox proportional hazards regression			
Score		Unadjusted HR for progression (CI)	Adjusted^a^ HR for progression (CI)	Unadjusted HR for death (CI)	Adjusted^a^ HR for death (CI)
PS category	0	1	1	1	1
	1	1.01 (0.76, 1.33)	0.99 (0.75, 1.31)	0.92 (0.68, 1.25)	0.93 (0.68, 1.25)
	2	**1.44 (1.06, 1.95)**	1.31 (0.96, 1.78)	**1.46 (1.06, 2.01)**	1.33 (0.96, 1.85)
	>2	1.36 (0.74, 2.53)	1.27 (0.68, 2.40)	1.03 (0.51, 2.08)	0.96 (0.47, 2.00)
GO2FS category	Not frail (0–1)	1	1	1	1
	Frail (2)	1.11 (0.83, 1.50)	1.07 (0.79, 1.44)	1.09 (0.78, 1.51	1.09 (0.78, 1.52)
	Sev frail (≥3)	**1.47 (1.14, 1.90)**	**1.44 (1.11, 1.85)**^**b**^	**1.62 (1.23, 2.14)**	**1.66 (1.25, 2.20)**^**b**^
mCFS category	Fit (1–2)	1	1	1	1
	Pre-frail (3–4)	**1.54 (1.19, 2.00)**	**1.57 (1.21, 2.05)**^**b**^	**1.65 (1.25, 2.19)**	**1.71 (1.29, 2.28)**^**b**^
	Frail (5+)	**1.66 (1.34, 2.06)**	**1.63 (1.30, 2.03)**^**b**^	**1.72 (1.35, 2.18)**	**1.75 (1.37, 2.22)**^**b**^
G8 category	≤14 (‘abnormal’)	1	1	1	1
	>14 (‘normal’)	**0.63 (0.44, 0.90)**	**0.63 (0.44, 0.90)**^**b**^	**0.54 (0.35, 0.81)**	**0.54 (0.36, 0.82)**^**b**^
CARG toxicity risk category	Low (0–5)	1	1	1	1
	Med (6–9)	0.88 (0.61, 1.25)	1.13 (0.77, 1.66)	0.88 (0.60, 1.28)	1.17 (0.78, 1.77)
	High (10–19)	0.92 (0.64, 1.32)	1.25 (0.83, 1.86)	0.90 (0.62, 1.33)	1.26 (0.83, 1.92)

Statistically significant results in **bold**. a = adjusted for: age group, sex, histology, presence of metastases, planned use of trastuzumab, dose reduction due to renal/hepatic impairment. b = significant after adding in PS to adjustment factors.

Abbreviations: OTU = overall treatment utility, PS = Performance Status, GO2FS = GO2 Frailty Score, mCFS = ‘modified’ Clinical Frailty Scale, G8 = Geriatric-8, CARG = Cancer and Aging Research Group.

A performance status of 2 was associated with an unadjusted hazard ratio of 1.44 (1.06–1.95) for progression and 1.46 (1.06–2.01) for death, but the statistical significance did not remain after adjustment for covariates. Other PS scores did not have a statistically significant correlation with survival outcomes before or after adjustment.

High GO2FS and mCFS scores and low G8 scores were associated with a statistically significant increased risk of both progression and death. Adjusted hazard ratios for death were as follows: 1.66 (1.25–2.20) for GO2FS ≥3 (severely frail), 1.71 (1.29–2.28) for mCFS 3–4 (pre-frail), 1.75 (1.37–2.22) for mCFS 5+ (frail) and 0.54 (0.36–0.82) for G8 > 14 (‘normal’). All of these associations remained after adjustment, including for performance status. There was no statistically significant association between CARG score and survival outcomes, with adjusted HRs for progression of 1.13 (0.77–1.66) for medium risk (score 6–9) and 1.25 (0.64–1.86) for high risk (score 10–19) and similar HRs for the risk of death.

### 3.4 Adjustment for PS and test for co-linearity

Table 4, Table 5 give unadjusted and adjusted data. The presented adjusted ORs and HRs do not include adjustment for PS due to concerns about co-linearity. PS was included in the multivariable models for GO2FS/mCFS/G8/CARG alongside the other adjustment factors as an additional analysis, and if significance remained, as it did in most cases, this is indicated in the tables. Whilst PS adjustment was not included in the presented adjusted estimates due to concerns about co-linearity, VIF tests were undertaken on each model with the inclusion of PS and these scores were low (<1.3) in all cases (where 1 = no correlation and > 5–10 = high correlation) suggesting that even with the inclusion of PS, multicollinearity was not present in the models.

## 4 Discussion

### 4.1 Key Findings

This retrospective cohort study explored the correlation between frailty measures and treatment outcomes in the GO2 randomised controlled trial database. Higher GO2FS and mCFS scores and lower G8 scores were associated with a statistically significant increased risk of poor Overall Treatment Utility, progression and death. This persisted after adjustment for confounding factors, including PS, demonstrating that assessment of frailty adds value on top of PS. Higher PS and CARG scores were also associated with a higher risk of poor outcome, but this was not statistically significant (except pre-adjustment HRs for death and progression in PS2 patients, and post-adjustment ORs for poor OTU in the numeric CARG analysis and categorical CARG ‘high risk’ sub-group). It may be that this study was underpowered to detect a statistically significant difference in outcome for these measures. Hazard ratios for mortality for frail patients were comparable to those reported elsewhere in the literature [1].

### 4.2 Selection of Frailty Measures

The derivable measures selected for evaluation included a range of measures with a potential role in assessing older and frail younger patients prior to SACT. It is important to note that whilst the CARG score does assess some frailty domains (e.g. mobility/falls and IADLs), it is not a frailty assessment per-se and is designed to predict short-term toxicity rather than longer-term outcomes. Regardless, we felt it would be interesting and valuable to see whether this translated to predicting global outcomes. Unfortunately, there were a number of notable measures, including VES-13, the Edmonton frailty score and CRASH toxicity score, which we were unable to derive. It is also important to note that many of the measures analysed here are only validated in specific age groups e.g. >70 for G8 [9] and > 65 for CFS [4]. We opted to undertake all analyses on the whole cohort (including frail younger patients), treating all frailty measures the same as far as possible. This likely improved the power of the study to detect a statistically significant association between frailty and outcome, but could have caused under-estimation of the association between frailty and outcome in older patients. Significant associations were detected despite inclusion of all ages. Further study to validate frailty measures in younger cohorts is warranted.

### 4.3 Implications of the Prognostic Capacity of Frailty Measures

Performance status was not associated with outcome in this analysis, further highlighting its shortcomings as a measure of fitness to guide treatment decision-making in oncological practice. However, frailty (identified via a range of measures) is associated with poor treatment outcome, and that association persisted after adjusting for PS in most cases, supporting the notion that frailty measures offer additional value to PS in assessing fitness for and weighing risks and benefits of SACT [2,3,6,7,11]. The correlation of frailty measures with OTU, a global patient-centred measure of treatment outcome, highlights that of assessment of frailty has prognostic value beyond traditional survival outcomes, further emphasising their potential role of frailty assessment in holistic decision-making. The question also remains as to whether the assessment of frailty and understanding of the association between frailty and outcome can be used to inform discussions and shared decision-making alongside patients. In the initial GO2 study analysis [13], a univariate analysis was undertaken to identify variables which predicted OTU; frailty (via GO2FS) was one of three variables that were independently predictive of OTU (alongside neutrophil: lymphocyte ratio and baseline global self-rated health via ED-5D). A multivariable model was created whereby each of these three variables can be fed in to provide estimates for the probability of a good, intermediate and poor OTU. Further study could look to further validate this model and explore whether GO2FS could be replaced with a simpler measure of frailty (e.g. CFS or G8) to improve the acceptability and feasibility of applying the model in clinical practice, as a clinical and/or patient decision-aid.

Frailty assessments are not widely undertaken in clinical trials or clinical practice, but these findings add weight to the argument that they should be integrated into routine practice [8,15]. The fact that the findings are relatively consistent across a range of frailty measures demonstrates that any measure of frailty likely has some value in decision-making, but for widespread use a measure would need to be both validated in this setting and feasible to undertake in day-to-day practice. A key strength of G8 is that it has been specifically developed and validated in the cancer setting and is recommended in international guidelines [8]. It can be undertaken with relative ease, but does still require the completion of a questionnaire which can be a barrier to use in a busy clinical environment. In contrast, CFS has been developed and widely tested in other settings [[5], [6], [7]], but despite its increasing interest in cancer care, it is not yet validated in cancer settings. However, it can be undertaken as part of the routine assessment of a patients' symptoms, functioning and social circumstances with no additional paperwork or assessments required, which gives it great potential for implementation within the busy clinic setting. We feel its simplicity and cross-speciality use make it a potentially fantastic tool for providing a common language for assessment and communication of frailty, spanning all specialities and professionals involved in cancer care, but further validation in the cancer setting is required to support its use.

### 4.4 Assessment and Retrospective Derivation of Frailty in Cancer Research

This study provides proof of concept for the ability to derive a global measure of frailty such as mCFS retrospectively from questionnaire responses in existing datasets such as clinical trials, which and could prove valuable for studying frailty. Given that frailty is clearly a confounder to cancer treatment outcome, we propose that frailty should be assessed at baseline and reported alongside demographic data in cancer research (including observational studies and clinical trials), to provide an understanding of the extent and outcomes of frailty within cancer cohorts. However, in the absence of prospective frailty assessment, retrospectively derived measures could have a role and where the required data exists, the R code created for this analysis could be applied to other datasets with relative ease. For example, mCFS could be derived from other datasets that have collected EORTC QLQ-C30 (for mCFS 1–4), IADL (for CFS 5–6) and EQ-5D (for mCFS 7) and work is going to identify potential datasets in which to test the algorithm. Moving forward, international consensus for the measurement and reporting of frailty in clinical trials would be valuable.

### 4.5 The mCFS Algorithm and CFS

The retrospective derivation of mCFS in this analysis is a key limitation of this study. In developing the algorithm to derive mCFS, it was notable that the baseline questionnaires did not perfectly align with the Rockwood CFS score descriptors and there was some subjectivity when selecting questions and responses to discriminate between CFS scores, which highlights limitations of retrospective derivation. However, we aimed to overcome this by developing a ‘best fit’ algorithm, recognising that mCFS is a proxy rather than a perfect measure of CFS, and used external review to help overcome individual bias. Further development of the mCFS algorithm through more rigorous methodology, such as a formal Delphi consensus study, may help to improve the validity of the measure. Whilst we do not know how well mCFS derived from the baseline questionnaires in this study correlates with clinician-assessed CFS in the clinic, there is some evidence demonstrating good inter-rater reliability between prospective and retrospective CFS assessment [18], which supports the validity of the findings of this study. Whilst this study demonstrates the potential prognostic value of the simple CFS assessment, further study looking at the association between prospectively-assessed CFS and treatment outcome is required before CFS scores can be used to guide decision-making in the clinic, and publication of existing data from the SCFN pilot sites [5] that have been collecting CFS in cancer settings could be valuable in this regard. A small prospective study looking at the agreement between mCFS and CFS could also be valuable.

## 5 Conclusion

Frailty, identified by three derivable measures (G8, GO2FS and mCFS), correlates with poor Overall Treatment Utility, progression, and death in this cohort of older and frail younger patients with aGO cancer. Frailty measures add information over and above PS, adding weight to the argument that assessment of frailty should be integrated into routine clinical and research practice in oncology. The simple measure of CFS has predictive potential in this setting that is similar to other more complex tools, though prospective study is required to corroborate the findings of this study. We have demonstrated that frailty measures can be derived retrospectively from existing data, with the potential to help to build the evidence base around the optimal care of frailer patients in the absence of prospectively collected frailty measures.

## Ethics approval

The GO2 trial (ISRCTN44687907) was approved by the UK National Research Ethics Service and overseen by independent Trial Steering and Data Monitoring & Ethics Committees.

## Consent for publication

All authors provide consent for publication.

## Availability of data and materials

De-identified participant data will be made available upon request. Any requests for trial data and supporting material (data dictionary, protocol, and statistical analysis plan) will be reviewed by the trial management group in the first instance. Only requests that have a methodologically sound proposal will be considered. Proposals should be directed to the corresponding author in the first instance; to gain access, data requestors will need to sign a data access agreement.

## Funding

The GO2 trial was funded by Cancer Research UK (trial number: CRUK/12/022) and ran within the UK National Health Service. Dr. Jessica Pearce, Academic Clinical Fellow [ACF-2019-02-006], is funded by Health Education England (HEE) / National Institute for Health Research (NIHR) for this research project. Dr. David Cairns is supported by Core Clinical Trials Unit Infrastructure from 10.13039/501100000289Cancer Research UK [C7852/A25447]. Dr. Mark Baxter is a Clinical Academic Fellow funded by the Scottish Chief Scientist Office. Funders had no role in designing, undertaking or reporting the study and the views expressed in this publication are those of the author(s) and not necessarily those of the funders, NHS or the UK Department of Health and Social Care.

## Author contributions

Conception and Design: JP, DS, GV and SN.

Data Collection: GO2 investigators.

Analysis and Interpretation of Data: JP, MB, DC.

Manuscript Writing: JP.

Approval of Final Article: All authors.

## Declaration of Competing Interest

Dr. Petty reported personal fees from Eli Lilly, Bristol Myers Squib, and Servier, and grants from AstraZeneca, Roche, Sanofi, Merck Sharp & Dohme, Five Prime Therapeutics, and Jansen outside the submitted work. Prof Seymour reported grants from Cancer Research UK during the conduct of the study. No other disclosures were reported. Dr. Hall reported grants from Cancer Research UK during the conduct of the study and institutional research funding from Novartis, Pfizer, Eli Lilly, Daiichi-Sanchyo, and Eisai outside the submitted work. Prof Velikova reported personal fees from Roche, Eisai, Novartis, and Seattle Genetics, and grants from Breast Cancer Now, European Organisation for Research and Treatment of Cancer, Yorkshire Cancer Research, and Pfizer outside the submitted work.

## Appendix A Supplementary data

Supplementary material 1 Image 1

Supplementary material 2 Image 2
