# Supplementary material for: Frailty and treatment outcome in advanced gastro-oesophageal cancer: An exploratory analysis of the GO2 trial
Source: J Geriatr Oncol. 2022 Apr;13(3):287–93. doi: 10.1016/j.jgo.2021.12.009 (PMC8986151; doi:10.1016/j.jgo.2021.12.009)
Supplement: Supplementary file 1 — Supplementary material 1 [file mmc1.docx]

**Supplementary appendix A: ‘Modified’ Clinical Frailty Scale Algorithm**

This algorithm has been developed to derive a ‘modified’ Clinical Frailty Scale (CFS) score from the baseline questionnaire data for patients in the GO2 trial (doi:10.1001/jamaoncol.2021.0848), as part of an exploratory analysis looking at the correlation between frailty measures and treatment outcomes.

The questionnaire data used to derive this ‘modified’ CFS include:

- Instrumental Activities of Daily Living (IADL) – available at: <https://www.bgs.org.uk/sites/default/files/content/attachment/2018-07-05/lawton_brody.pdf>
- Quality of life questionnaire EORTC QLQ-C30 – available at: <https://www.eortc.org/app/uploads/sites/2/2018/08/Specimen-QLQ-C30-English.pdf>
- EQ-5D-3L – available at: <https://euroqol.org/wp-content/uploads/2020/09/Sample_UK-English-EQ-5D-3L-Paper-Self-Complete-v2.1-ID-23963.pdf>

The algorithm aims to provide a ‘best fit’ for placing patients within the CFS scores and descriptors described in Rockwood CFS v2 (doi:10.5770/cgj.23.463), based on the available questionnaire data. The algorithm was drafted by Drs Jessica Pearce (Medical Oncology trainee), Sherena Nair (Elderly Medicine consultant) and Daniel Swinson (Medical Oncology consultant) and revised in line with feedback following independent review by two other Elderly Medicine consultants. Future work building on from this may involve a formal Delphi consensus study and/or external validation in other datasets.

| **Rockwood Clinical Frailty Scale v2.0**  **Score and descriptor** | **Corresponding criteria from baseline questionnaire** |
| --- | --- |
| **CFS 1: Very fit**  Very Fit – People who are **robust, active**, energetic and motivated. They tend to exercise regularly and are among the **fittest for their age**.  *Key points:*  *-Fittest for their age – no trouble with long walk/strenuous activity* | Meets ALL of the following criteria:   - Able to do **strenuous activities** AND **long walk** without trouble (EORTC QLQ-C30 Q1-3 answer 1)   [AND none of the CFS 4/5/6/7 criteria] |
| **CFS 2: Well**  People who have no active disease symptoms but are **less fit than category 1**. Often, they exercise or are very active occasionally, e.g. seasonally.  *Key points:*  *-Well but less fit than CFS 1 i.e. can do a short walk without trouble but may have trouble with long walk/strenuous activity* | Meets ALL of the following criteria:   - Able to do **short walk** without trouble (EORTC QLQ-C30 Q3 answer 1)   [AND none of the CFS 1 or 4/5/6/7 criteria] |
| **CFS 3: Managing well**  People whose medical problems are well controlled, even if occasionally symptomatic, but are not regularly active beyond routine walking.  *Key points:*  *-Lack of CFS 4 criteria demonstrates medical problems being well controlled*  *-Lack of CFS 5-7 criteria demonstrates that they are reasonably independent*  *-‘Not regularly active beyond routine walking’ reflected in the fact they don’t fulfil CFS 1/2* | DOES NOT meet any of the criteria for:   - CFS 1-2 OR - CFS 4-7 |
| **CFS 4: Living with very mild frailty**  Previously “vulnerable”, this category marks early transition from complete independence. While **not dependent on others for daily help**, often **symptoms limit activities**. A common complaint is being **“slowed up”**, and/or being **tired** during the day.  *Key points:*  *-Lack of CFS 5-7 criteria demonstrates not dependant*  *-Criteria differentiating from CFS3+ are feeling tired and symptoms limiting activity* | Meets ANY of the following criteria   - *In the last week…* **feels tired** quite a bit or very much (EORTC QLQ-C30 Q18 answer 3 or 4)   *OR*   - *In the last week…* physical condition or medical treatment **interferes with family life or social activities** quite a bit or very much (EORTC QLQ-C30 Q26+27 answer 3 or 4)   [AND none of the CFS 5/6/7 criteria] |
| **CFS 5: Living with mild frailty**  People who often **have more evident slowing**, and need **help** in high order IADLs  (**finances**, **transportation**, **heavy housework**). Typically, mild frailty progressively impairs **shopping** and **walking outside alone**, **meal preparation, medications** and begins to restrict light **housework**.  *Key points:*  *-More evident slowing i.e. in bed/chair quite a bit*  *-Impaired ADLs/needing help* | Meets ANY of the following criteria:   - Has to stay in bed or a chair during the day quite a bit or very much (EORTC QLQ-C30 Q.4 answer 3-4) - Needs to be accompanied for any shopping trip (IADL Q.B answer 3) - Needs to have meals prepared and served (IADL Q.C answer 4) - Performs light daily tasks but cannot maintain acceptable level of cleanliness – or worse (IADL Q.D answer ≥3) - Travel limited to taxi or automobile with assistance of another (IADL Q.F answer ≥3) - Takes responsibility [for medication] if medication is prepared in advance in separate dosage - or unable (IADL Q.G answer ≥2) - Manages day-to-day finances but needs help with banking, major purchases, etc. or completely incapable of handling money (IADL Q.H answer ≥2)   [AND none of the CFS 6/7 criteria] |
| **CFS 6: Living with moderate frailty**  People need **help with all outside activities** and with **keeping house**. Inside, they often have **problems with stairs** and **need help with bathing** and might need minimal **assistance (cuing, standby) with dressing**.  *Key points:*  *-Needs help all outdoor activities (shopping and transport) and with more basic/fundamental ADLs* | Meets ALL of the following criteria:   - Needs to be accompanied on any shopping trip - or completely unable to shop (IADL Q.B. answer ≥3)   AND   - Travels on public transport when accompanied by another - or limited to taxi/automobile with assistance - or does not travel at all (IADL Q.F ≥3)   AND  Any one of the following:   - Needs quite a bit/very much help with eating, dressing, washing or using toilet (EORTC QLQ-C30 Q.5 answer 3-4) - Performs light daily tasks but cannot maintain acceptable level of cleanliness – or worse (IADL Q.D answer ≥3) - Is not capable of dispensing own medications (IADL Q.G answer 3)   [AND none of the CFS 7 criteria] |
| **CFS 7: Living with severe frailty**  **Completely dependent for personal care**, from whatever cause (physical or cognitive). Even so, they seem stable and not at high risk of dying (within ~ 6 months).  *Key points:*  *-Completely dependant* | Meets the following criteria:   - Unable to wash or dress self (EQ-5D Q2 [SELFCARE] answer 3) |
| **CFS 8: Living with very severe frailty**  Completely dependent, approaching the end of life. Typically, they could not recover even from a minor illness. | - Not applicable (there are no questions in GO2 baseline data that would differentiate CFS 8 and it would not be expected in patients included in the GO2 trial) |
| **CFS 9: Terminally ill**  Approaching the end of life. This  category applies to people with a life expectancy <6 months, who are not otherwise living with severe frailty. (Many terminally ill people can still exercise until very close to death.) | - Not applicable (there are no questions in GO2 baseline data that would differentiate CFS 8 and it would not be expected in patients included in the GO2 trial) |

The algorithm was created in the open-source coding platform Rstudio. Please get in touch if you would like to collaborate on further development of the algorithm and/or testing the algorithm in your dataset [j.pearce@leeds.ac.uk](mailto:j.pearce@leeds.ac.uk) (Jessica Pearce).
