# Supplementary material for: Frailty and treatment outcome in advanced gastro-oesophageal cancer: An exploratory analysis of the GO2 trial
Source: J Geriatr Oncol. 2022 Apr;13(3):287–93. doi: 10.1016/j.jgo.2021.12.009 (PMC8986151; doi:10.1016/j.jgo.2021.12.009)
Supplement: Supplementary file 2 — Supplementary material 2 [file mmc2.docx]

**Supplementary appendix B: Overall survival curves, stratified by each frailty measure**


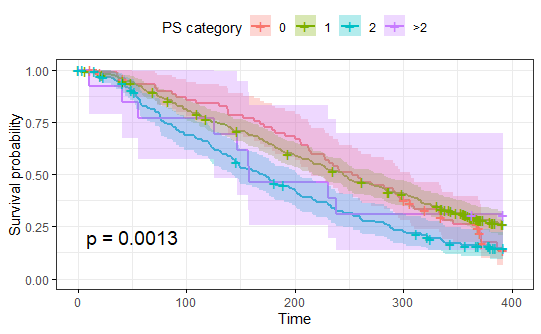

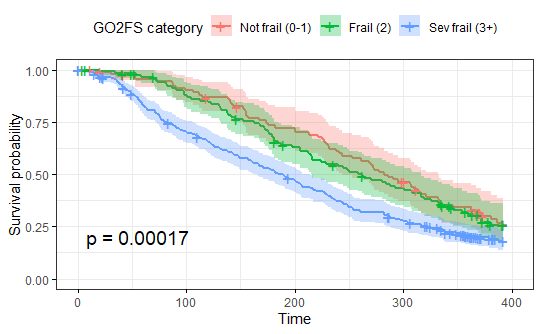

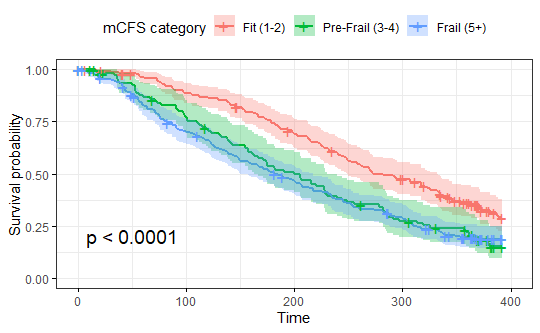

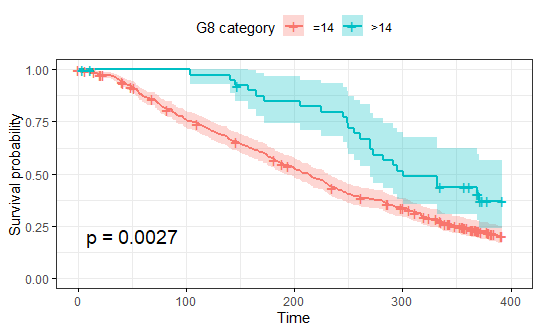


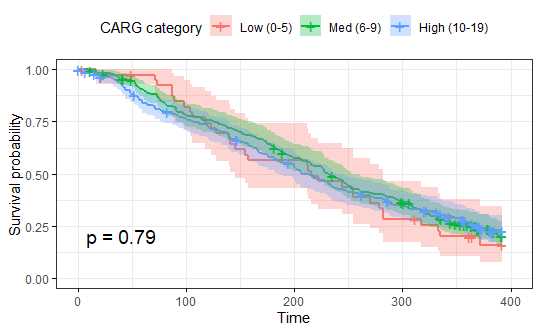


Abbreviations: PS = Performance Status, GO2FS = GO2 Frailty Score, mCFS= ‘modified’ Clinical Frailty Score, G8 = Geriatric-8, CARG = Cancer and Aging Research Group.
